# Long-term members’ use of fitness centers: a qualitative study

**DOI:** 10.1186/s13102-019-0114-z

**Published:** 2019-02-21

**Authors:** Liv Riseth, Torunn Hatlen Nøst, Tom I. L. Nilsen, Aslak Steinsbekk

**Affiliations:** 1 Department of Public Health and Nursing, Norwegian University of Science and Technology, P.O.Box 8905, 7491 Trondheim, Norway; 2 3T- Fitness Center, Vestre Rosten 80, 7075 Tiller, Norway; 3 Clinic of Anaesthesia and Intensive Care, St Olavs Hospital, Trondheim University Hospital, Trondheim, Norway

**Keywords:** Fitness center, Physical activity, Long-term members, Qualitative methods

## Abstract

**Background:**

Although the health benefits of physical activity are well documented, a large proportion of the population remains less active than recommended by current guidelines. Commercial fitness centers provide an opportunity to perform physical activity and exercise, but there has been little research focusing on ordinary members at commercial fitness centers. The aim of this study was therefore to explore what long-term members (> 2 years) wanted to achieve with their membership and to identify important factors that influenced them to use the fitness center as a means for physical activity.

**Method:**

This was a qualitative study with 21 semi-structured individual interviews of adult long-term fitness center members in Trondheim, a city in Central Norway with approximately 190,000 inhabitants. The participants had been continuous fitness center members for more than two years and were asked about their experiences using a fitness center and what they wanted to achieve with the membership. The data was analyzed thematically with the method of systematic text condensation.

**Results:**

The results were categorized into three main themes: “Health benefits and physical appearance”; “Accessible, safe, and comfortable to use”; and “Variety, flexibility, and support.” The participants stated that they wanted to achieve health benefits, but they also talked about physical appearance. The fitness center was mainly described as easily accessible and a comfortable place for physical activity. Some female participants emphasized the feeling of safety compared to outdoor activity. Variation in activities, making commitments, and getting support from staff and other members were factors contributing to use of the fitness center for physical activity.

**Conclusion:**

Achieving desired health benefits and improving physical appearance were the main drivers for long-term members’ use of the fitness center. The fitness center was preferred due to the comfort of the facilities and the possibility to commit to specific exercise times and activities.

## Background

The effects of physical activity to improve or maintain good health are well documented [1]. Despite public health recommendations and encouraging advice to stay physically active [2, 3], approximately 30% of adults worldwide are physically inactive [4]. Physical activity behavior depends on a multitude of barriers and facilitators [5], such as accessibility [6–8], weather [9], and social support [10, 11]. In addition, it includes many different psychological components, such as habits [12, 13], planning [14–16], perceived behavioral control [17], motivation [18], physical activity identity [17], personality [19], and self-efficacy [16, 17]. Due to the high prevalence of physical inactivity, increased levels of physical activity are a global public health priority [20].

Commercial fitness centers represent one opportunity to be physically active. The majority of fitness centers offer both group and individual activities [21, 22]. In recent years, there has been an increase in the proportion of people who attend commercial fitness centers, with 15% of the European adult population doing so in 2013 [23]. This number is higher in some countries; for instance, it is 19.4% in Norway [24]. Statistics show a steady increase in attendance at private fitness centers in European countries; the number of members was 56.4 million at the end of 2016, which was an increase of 4.4% from the previous year [24]. Thus, fitness centers are an important arena for physical activity. However, it has been found that members would like to exercise more regularly than they do [25].

Former studies among fitness center members have focused on reasons to become and remain a member [25, 26] and have highlighted motivational differences between fitness center and sports club members, e.g., sports club members are reported to be more motivated by competition, pleasure, and social factors and less concerned with appearance than fitness center members [21, 27, 28]. It has also been found that women report a slightly higher desire for wellness, a well-trained body, and weight loss than men do, while men report a desire for improved physical fitness [22, 25, 27, 29, 30].

Although a large proportion of the population are fitness center members, research on exercise behavior of members in fitness centers is limited in quantity and quality [31]. Therefore, it is important to gain in-depth knowledge of ordinary members (those who pay regular membership fees). Long-term members are an especially interesting group as they might have found a way to use the fitness center to achieve their goals for physical activity over time. However, we have not found any studies focusing exclusively on long-term members.

The aim of this study was therefore to explore what long-term members (> 2 years) wanted to achieve with their membership and to identify important factors that influenced them to use the fitness center as a means for physical activity.

## Methods

This was a qualitative study with semi-structured face-to-face individual interviews conducted between March 2015 and November 2016.

### Setting

In Norway, with 5.2 million inhabitants, it is estimated that there are 1079 fitness centers [24]. Within physical activity, there is also a strong tradition for participating in voluntary organizations in Norway, where sports clubs constitute the largest proportion [32].

This study took place among members of different fitness centers in Trondheim, a city in Central Norway with approximately 190,000 inhabitants, within the 3 T-Fitness Center chain (www.3t.no). 3 T established its first fitness center in 1985, and the chain is now the largest fitness center chain in Central Norway, with 16 fitness centers and approximately 40,000 members. During data collection, the fitness center chain had a total of 12 fitness centers, with eight of them located in Trondheim. A membership in this fitness center chain has a cost of 350–550 NOK (approximately 40–60 EUR) per month. To become a member, one has to sign a one-year contract; thereafter, one can terminate the contract one month after giving notice. The centers in this chain have a diversity of training opportunities, with a main workout area that primarily consists of free weights, weight machines, rowing machines, stationary exercise bikes, elliptical trainers, and treadmills. Nearly all centers offer group classes, personal trainers, saunas, and member lounges with simple café services. Some centers offer squash, childcare, and physical therapy. The largest center also has a swimming pool and a wellness area.

### Participants

The inclusion criteria for this study required that each participant was older than 18 years and had been a paying member for more than two years continuously (to avoid the large proportion terminating their membership after the obligatory one-year contract) at one of the eight 3T fitness centers in Trondheim. We sought a sample with diversity in gender, age, frequency of visits, and types of services used, ranging from those included in the membership to services with extra cost such as hiring a personal trainer.

The first step in recruiting participants consisted of randomly selecting from the membership register 24 members who met the inclusion criteria. Each of these prospects received a letter or an email with information about the study and a request to participate. The message also stated that they would be contacted by phone after two weeks if they did not respond before that time. Of these 24, 16 responded positively and were interviewed. However, none of these participants were under the age of 27, nor had they used additional services with extra cost. Therefore, eight additional prospects were identified in the member register or by employees at the fitness center. They were contacted as described above, resulting in two more participants who were in their early 20s and three participants (age range 30–57 years) who had used services with extra cost (personal trainer, physiotherapist, and / or nutrition supervisor).

### Data collection

The interviews were conducted by the first author at a place chosen by the participants and lasted from 32 to 62 min (average 48 min). The interviews were audiotaped and transcribed verbatim.

An interview guide with open-ended questions was developed by the first author, based on former literature about fitness center use, goal achievement, motivation, and the patient experiences questionnaire (PEQ) [26, 29, 31, 33–35], as well as discussions among the authors and a research group three of the authors belong to. The four main questions were: “Can you tell me about your experiences of being a member at the fitness center?”, “What contributes to your use of the fitness center?”, “What do you want to achieve with the membership?”, and “Is there anything the fitness center can do to make it easier for you to achieve what you want with the membership?”. The following topics were introduced if the participant did not spontaneously talk about them: description of your own physical activity behavior; what affects your use of the fitness center (facilities, opening hours, activities available, social interactions, family, friends, and support and presence of staff); and reasons why you achieve / do not achieve what you want with the membership.

### Data analysis

The data was analyzed thematically with the method of systematic text condensation (STC) by Malterud, which is inspired by Giorgi’s psychological phenomenological method [36]. An illustration of the STC process and how the participant responses were coded and categorized is given in Table 1. The STC process is an iterative four-step method suitable for descriptive cross-case analysis of qualitative data. The method was chosen because it is well suited to present participants’ experiences, rather than the possible underlying opinions of what was told [36]. All interviews were held in Norwegian and the material was kept in its original language throughout the analysis. The data analysis was conducted by the first author in cooperation with the co-authors and discussed twice in an established research group.

**Table 1:** Illustration of the steps in STC

Step 1: Preliminary theme	Step 2: Meaning units (direct citation from informant)	Step 3: Condensed description	Step 4: Final theme and analytical text
Achieves physical and mental health benefits	“The important thing for me is that I manage to avoid another operation on my knee. Water gymnastics is good for me and I want to continue with this in the future.” “Yes, I feel the positive effects exercise has for me, in relation to both energy and my mood. And my anxiety, it affects that a lot. I have had years where I only have been sitting indoors, without getting out of the house”	Exercise is positive in relation to my health; it also leads to more energy and better mood.	Theme: Health benefits and physical appearance Analytical text: The fitness center was […] and to achieve desired health benefits.

In the first step of the analysis, the first author and the co-authors read the transcripts from a bird’s-eye perspective to identify preliminary themes. In the second step, meaning units (text segments) relevant to the aim of the study were identified by the first author, then coded and sorted into code groups based on the preliminary themes. The second step was done repeatedly, with several meetings and discussions among co-authors and the research group. MindManager [37] was used as a systematization tool in this part of the analysis. In the third step, a condensed description of the citations in each code group was made using the participants’ original phrases. Finally, in the fourth step, the descriptions were rephrased into analytical text.

The first sequence of analysis included the first thirteen interviews and was performed to identify areas that needed to be explored in more detail in further interviews. This was done because analyzing the data stepwise may contribute to systematic improvement of data collection, facilitate reflection, and reduce the number of participants needed [36, 38]. The four steps of the STC were performed repeatedly in both the preliminary stages and the final analysis, leading to several changes and modifications before the final themes were agreed upon.

The analysis was validated by continuously checking the findings against the transcripts, especially after the final analysis. The first author identified illustrative citations and discussed them with the co-authors to choose the ones that best illustrated the themes. These were translated from Norwegian to English by the first author and checked by the other authors.

## Results

A total of 21 long-term members (11 females and 10 males) from eight different fitness centers were interviewed (Table 2). They had been members for 2–20 years and their average age was 43 years (range 20–71 years).

**Table 2:** Demographic characteristics of Participants and members at the fitness centers who have been continuous members for at least two years

Characteristics	Participants N (%)	All similar members at fitness center chain %*
Gender		
- Female	11/53%	59%
- Male	10/47%	41%
Age		
- 20–29	6/28.5%	26%
- 30–49	9/43%	45%
- 50–71	6/28.5%	22%
Duration of membership		
- 2–5 years	9/43%	Median: 2–5 years
- 6–10 years	8/38%	
- > 10 years	4/19%	
Number of visits last two years		
- < 9 visits	1/5%	9%
- 10–49 visits	8/38%	35%
- 50–100 visits	8/38%	24%
- > 100 visits	4/18%	32%
Highest level of education		
- Primary school / Lower secondary school	1	
- Upper secondary school	5	
- Some college / university courses	4	
- Bachelor’s degree	11	
- Graduate degree or Advanced degree	2	
Employment status		
- Student (university) full time	1	
- Student and working part time	4	
- Working part time	4	
- Working full time	8	
- Receiving disability benefits or other auxiliary benefits	2	
- Retirement pension	1	

* All paying members from November 2014 to November 2016 at 3 T-Fitness Center

What long-term members wanted to achieve with their membership is described in the theme “Health benefits and physical appearance.” Their experiences with factors affecting their use of the fitness center as a means for physical activity were categorized into the themes “Accessible, safe, and comfortable to use” and “Variety, flexibility, and support.”

### Health benefits and physical appearance

The long-term members said that they used the fitness center to achieve desired health benefits. The main examples of health benefits were more energy, improved mood and sleep, reduced stress, better well-being, or feeling happier after the workout. Some participants with health complaints said that use of the fitness center was a necessity for their daily function, e.g., to prevent deterioration of their health complaint, and as an aid in pain management.

*Yes, I feel the positive effects exercise have for me, in relation to both energy and my mood. And my anxiety, it affects that a lot. I have had years where I only have been sitting indoors, without getting out of the house. (Female, 30–49 years, member for 8 years).*

However, during the interviews some long-term members mentioned that they wanted to achieve a better look. Physical appearance was seldom directly talked about as a reason. The participants rather mentioned it in passing, e.g., adding it after having talked about another reason, followed by a joke or laughter. The things they said that concerned physical appearance included a fear of becoming overweight, wanting to get into the clothes they had worn before, reducing abdominal size, and becoming more muscular.

*I have never been concerned about how I look and exercise solely in order to be able to continue to work and avoid a new knee replacement. But I should perhaps have reduced this slightly [laughs and pats the stomach]. (Male, 50–71 years, member for 4 years).*

Male participants, in particular younger males, talked more about wanting to achieve a muscular body and becoming stronger, while female participants talked more about weight loss. When asked about the reason for their focus on physical appearance in relation to the use of the fitness center, some female participants talked about increased emphasis in society on being thinner and fit.

*It feels like we should have been a little leaner, yes actually a little better at everything. If you do not exercise and stay healthy and slim, you are almost a bit questionable. According to everything that is communicated from the fitness center and health authorities (short pause). I do not like that it affects me so much. (Female, 30–49 years, member for 6 years).*

### Accessible, safe, and comfortable to use

The fitness center was primarily described as a comfortable place to be physically active. It was common to talk about physical activity in the fitness center as easier than being active outdoors. Due to the indoor comfort, avoiding bad weather and winter darkness, they said the fitness center made it easier to motivate themselves to be physically active. Living or working near the fitness center, ample opportunities for parking, and public transport were also given as reasons. Some female participants emphasized the importance of increased security when using the fitness center for physical activity compared to exercising outdoors on their own.

*I feel safe when I visit the fitness center and it is cozy getting inside to the reception area with those flames from the fireplace and the friendly staff in the reception. I am afraid of the dark and yes, I am a little afraid of being assaulted by someone when running outside. (Female, 20–29 years, member for 3 years).*

Even though some participants talked about how they enjoyed using the fitness center for physical activity, they described different challenges and barriers that hindered them in using the fitness center as much as they wanted, e.g., too little time, no childcare, and low motivation to get out of the house. Participants with children living at home especially expressed difficulties finding time and energy. It was said that having commitments such as appointments with others, pre-booked activities, or payment of no-show fees could help them to prioritize physical activity at the fitness center.

*It is a bit odd because I like it when I am at the fitness center, but sometimes or actually quite often, it is hard to get out of the door at home. It is very positive that one must pay if one does not meet for (pre-booked) group classes, because then I have to go. (Female 20–29 years, member for 2 years).*

Payment of membership fees and fees for working with personal trainers were said to affect use of the fitness center for some long-term members; typically, they wanted to use the services they had paid for. However, most participants stated that the fee was not something they thought of. One participant who was receiving disability benefits talked about the fitness center as an affordable option for exercise and as the only possibility to be regularly physically active.

*It is cheap for me to be a member of the fitness center and it means a lot to me, since I have limited money available. I do not have much money to spend when all expenses are paid, but it is working fine for me to pay a small amount each month [to be a member of the fitness center]. (Female, 50–71 years, member for 4 years).*

Some of the long-term members said that they valued the fitness center as a social meeting place where they could spend time with friends, family, and colleagues and make new acquaintances. This was facilitated by a friendly atmosphere at the fitness center and services like the opportunity to buy a cup of coffee and sit down for a chat.

*I appreciate having the opportunity to socialize with my friend and that we can sit down, relax, and have a chat after the workout. In fact, I always work out with someone. It's social. (Male, 50–71 years, member for 9 years).*

### Variety, flexibility, and support

Variety, with both group classes and various possibilities for self-training, was said by some long-term members to be important with regard to regular use of the fitness center for physical activity. Those using mainly group classes or self-training had different explanations for their use of the fitness center facilities as a means for physical activity.

A typical argument given for participating in group classes was as a help to exercise more vigorously compared to self-training. Some also said that they preferred group classes because they were time limited and followed a fixed structure. Furthermore, some spoke about the boost they got from the atmosphere and enthusiasm in the group classes. This was also mentioned as a reason for going to the fitness center in general, since it required less self-motivation. Other things mentioned were enjoyable experiences, being in a group with others, mastering a new step in a choreography, or a pleasant conversation.

*As an example, if I had chosen a fitness center without classes, instructors, or those types of facilities, I would have needed the inner motivation for exercising, and to be honest — that is not strong enough. I need something and someone to motivate me. (Male, 30–49 years, member for 5 years).*

The reasons for preferring self-training were described somewhat differently from group classes. The arguments given for self-training were flexibility, an all-in-one-place access to equipment, exercise at their own pace, and opportunities for targeted exercise in both strength and endurance. On the other hand, participants who did not perform self-training regularly spoke about challenges to implement it, e.g., because they found it boring, a duty, and not enjoyable.

*I have got an exercise program; everything else is too hard, group classes and such. I take my program at my own pace and it strengthens me. It is a bit similar to what I do with the physiotherapist, but here I do not have to see so many sick people. (Female, 50–71 years, member for 4 years).*

Some long-term members spoke positively about the help and the individual instructions they received from staff in the main workout area. It had helped them to understand what to do and how to do it when they used the fitness center. The staff were generally described as friendly, helpful, and knowledgeable if the members made appointments, but some perceived them as not very available for questions and help during workouts. Some participants had also used personal trainers and found it helpful for recognizing and understanding their physical capacity and getting a more efficient workout. Personal training was also described by some as helpful when implementing more regular use of the fitness center.

*Yes, I thought I was exercising. However, I realized with guidance from a personal trainer that I previously had been far from being able to call it exercise. So, everyone should try a personal trainer to really understand what one should do and how. (Female, 30–49 years, member for 15 years).*

## Discussion

The participants stated that they wanted to achieve health benefits, but they also talked about physical appearance. The fitness center was mainly described as easily accessible and a comfortable place for physical activity. Some female participants emphasized the feeling of safety compared to outdoor activity. Variation in activities, making commitments, and getting support from staff and other members were factors contributing to using the fitness center for physical activity.

There was a duality in what the long-term members wanted to achieve, between health benefits and appearance. The participants talked about the health benefits of physical activity as a main reason to use the fitness center. A survey has also found that members of fitness centers in Norway report they exercise to become fit rather than to gain a better-looking body [27], and the author suggested that it might feel better to say they exercise for fitness rather than for appearance. Similarly, in our study, appearance was mentioned as an additional reason after talking about health benefits. However, findings in another survey among fitness club members showed that seeing physical change as “becoming stronger” or “being able to see improvement in the way I look” were the main reasons for being physically active across age and gender [29].

This raises a question on whether long-term members of fitness centers are more occupied with their appearance than others doing physical activity in other settings. One study found this to be the case, with members of sports clubs being less concerned with appearance than fitness center members [27]. Similarly, a study on college students reported that those who engaged in exercise (e.g., aerobics, cycling, weight training) were more focused on appearance than those engaging in sports (e.g., tennis, basketball, soccer) [28]. It may be positive for fitness center members to focus on looks if it leads to a physically active lifestyle. However, for some, the focus on appearance might lead to a negative attitude towards oneself and make one exercise excessively [39–41]. It is therefore reasonable to question whether fitness centers should focus on appearance when marketing and promoting activities at the centers. In Norway, the main enterprise federation for fitness centers encourages them to be aware of the risk of excessive exercise in relation to disordered eating among their members.

Some of the participants said that they did not visit the fitness center as often as they wanted. A Danish report also found that most members of fitness centers would have liked to work out more frequently than they did [25]. An interesting finding in our study was that having committed to exercise through actions like pre-booking an activity, making a binding agreement with others, or making a payment was said to be the pressure they needed to visit the fitness center. This has not previously been reported, although it has been shown that making personal commitments using commitment devices with rewards or punishments for success or failure has been beneficial for behavioral changes [42, 43]. This can indicate that binding agreements such as pre-booking of activities, no-show fees, appointments, and even payments can be tested by the fitness centers to see if they promote increased use and consequently more regular physical activity among the members.

According to some participants in the present study, an important motivational factor for using the fitness center was the opportunity for social interaction. Participants reported benefits from social support from employees, group classes, and other members, but also from the possibility to be in a social setting. A review also concluded social support to be positively associated with levels of physical activity among adolescents [10], and another review on qualitative studies also found that development and maintenance of social support networks were important for participation in sport and physical activity [11]. Even if these reviews focus on physical activity in general, together with this study it is reasonable to hypothesize that fitness centers can help members to increase their motivation to use the fitness center if they facilitate more opportunities for social interaction. Moreover, a conscientious use of both social interaction and binding agreements might be even more helpful and motivating for some members.

As expected, all participants appreciated that the fitness center was easily accessible, safe, and a comfortable place to be, especially during the fall and winter months. It has also been suggested in a review that levels of physical activity vary with seasons [9]. On the other hand, one study found that weather showed a weaker relationship with physical activity than did accessibility [6]. Similarly, another study found that weather had modest effects on physical activity [44]. In general, easily accessible opportunities for physical activity are positively correlated with the level of activity [6–8]. Thus, in a public health perspective it is important to emphasize that having safe and easily accessible fitness centers might be a driver for more regularly physical activity in the population.

The factors identified in this study are based on experiences from long-term members who have chosen to continue as members for an extended period and naturally are quite satisfied with the fitness center as an arena for physical activity. However, the factors identified are most likely quite similar for all members, regardless of membership duration [26]. It is also possible that the identified factors are important factors generally to maintain physical activity over time.

### Strengths and limitations

A strength in the study is that it is the first to investigate what long-term members want to achieve with their membership and factors affecting their use of the fitness center as a means for physical activity. Another strength lies in the diversity of the sample. A limitation was that the study was done in a restricted geographical area and in only one fitness center chain. It also focused on long-term members and thus not those who for various reasons have terminated their membership. Moreover, it is possible that invited members who did not want to participate in the study are different from those who enrolled. Furthermore, younger participants were few in number. Younger participants might have had different experiences due to having other motives for participation in physical activity [45]. Given the similarity with findings in other studies on maintaining physical activity [26], it is not likely that the lack of younger participants has influenced the findings in the current study.

We consider it as a strength that the authors, who all took part in the analysis, have different backgrounds and experiences. Having researchers with other backgrounds, using a theory-driven approach, or doing member checking by inviting the participants to comment on the results could have produced other understandings and explanations.

At the time of the completion of the study, the first author worked in the administration of the fitness center chain, which might have influenced the research process. This was duly handled by having the co-authors participate in all steps of the research process, paying attention to the possibility of biases.

## Conclusion

This study indicates that the main drivers for long-term members’ use of a fitness center is to achieve desired health benefits and improve physical appearance. The prominent factors for using the fitness center were the comforts of the facilities and the ability to commit to exercise through fixed times for group activities, bookings, payments, and training agreements. Female members also valued the fitness center as a safe place for physical activity. Still, being physically active to the degree one wants is challenging, even for some long-term members.

From a public health perspective, the findings in this study point to commitment and having access to safe and easily accessible arenas for physical activity as being possible drivers for physical activity maintenance.

Further research is required to quantify the knowledge from this study. Doing a questionnaire-based survey with a randomly selected sample of fitness center users is recommended.

## Abbreviation

STC: Systematic text condensation
